# Comparison of HIV prevalence estimates from antenatal care surveillance and population-based surveys in sub-Saharan Africa

**DOI:** 10.1136/sti.2008.030106

**Published:** 2008-07-22

**Authors:** L S Montana, V Mishra, R Hong

**Affiliations:** 1 Harvard University, Boston MA, USA; 2 Macro International Inc, Calverton, Maryland, USA

## Abstract

**Objective:**

To compare HIV seroprevalence estimates obtained from antenatal care (ANC) sentinel surveillance surveys in Ethiopia, Kenya, Malawi, Tanzania and Uganda with those from population-based demographic and health surveys (DHS) and AIDS indicator surveys (AIS).

**Methods:**

Geographical information system methods were used to map ANC surveillance sites and DHS/AIS survey clusters within a 15-km radius of the ANC sites. National DHS/AIS HIV prevalence estimates for women and men were compared with national prevalence estimates from ANC surveillance. DHS/AIS HIV prevalence estimates for women and men residing within 15 km of ANC sites were compared with those from ANC surveillance. For women, these comparisons were also stratified by current pregnancy status, experience of recent childbirth and receiving ANC for the last birth.

**Results:**

In four of the five countries, national DHS/AIS estimates of HIV prevalence were lower than the ANC surveillance estimates. Comparing women and men in the catchment areas of the ANC sites, the DHS/AIS estimates were similar to ANC surveillance estimates. DHS/AIS estimates for men residing in the catchment areas of ANC sites were much lower than ANC surveillance estimates for women in all cases. ANC estimates were higher for younger women than DHS/AIS estimates for women in ANC catchment areas, but lower at older ages. In all cases, urban prevalence was higher than rural prevalence but there were no consistent patterns by education.

**Conclusions:**

ANC surveillance surveys tend to overestimate HIV prevalence compared to prevalence among women in the general population in DHS/AIS surveys. However, the ANC and DHS/AIS estimates are similar when restricted to women and men, or to women only, residing in catchment areas of ANC sites. Patterns by age and urban/rural residence suggest possible bias in the ANC estimates.

The HIV/AIDS epidemic is one of the largest public health crises of the 21st century. While the epidemic has spread over the past two decades, a cure or vaccine for HIV has remained elusive. The HIV prevalence estimates have come under increased scrutiny in recent years and some countries have revised their estimates downwards as more reliable data have become available. For example, the estimated number of HIV-infected people in India was revised downwards from 5.7 million to 2.5 million in 2007. Similar downward adjustments in HIV prevalence estimates have also been made for several countries in sub-Saharan Africa. As a consequence, UNAIDS and the World Health Organization have recently lowered the global estimate of the number of HIV-infected people from 39.5 million in 2006 to 33.2 million in 2007.^1^ While some imprecision in the global total may not make a substantial difference in international attention to the epidemic or resource allocations, the extent of imprecision may vary greatly by country and may have major consequences for the local public health response. Reliable data on HIV prevalence in the general population are essential for an effective response to the epidemic and its consequences.

Since the late 1980s, country-specific HIV prevalence estimates in countries with generalised epidemics have been derived from data collected at health facilities providing antenatal care (ANC) for pregnant women.^2^ Pregnant women are considered to be a good proxy for the general population, and this population is accessible through routine ANC visits, where blood is generally collected for other tests. However, HIV prevalence estimates based on pregnant women may be affected by biases, which can lead to overestimation of HIV prevalence among the general population.^3^ ^4^ Pregnant women are an imprecise proxy for the general population if pregnancy occurs more frequently at younger ages, and among rural, poorer women. Pregnant women are sexually active and may have been exposed to HIV, unlike their non-sexually active peers. HIV-infected women may be physiologically less likely to become pregnant, which can lead to an underestimation among women of same age in the general population. Furthermore, ANC coverage is not universal in all countries, the sites often cover a limited, more urbanised geographical area, and ANC data do not provide information on men.^5^

Given the increasing need for more precise data on the HIV epidemic, the population-based demographic and health surveys (DHS) began to include HIV testing of adult women and men in 2001. Population-based surveys have many advantages: they provide representative estimates for both women and men, for geographical regions and by age groups.^6^ Population surveys offer another significant advantage, the linkage of HIV status to individual respondent and household characteristics. The linked surveys allow for the analysis of behaviour, knowledge and background characteristics as they relate to HIV status. Since 2001, some three dozen population-based surveys with HIV testing have been or are being carried out under the DHS project.^7^

The purpose of this analysis is to quantify and interpret the differences between HIV prevalence estimates obtained from ANC sentinel surveillance surveys and from DHS/AIS surveys in selected countries in sub-Saharan Africa.

## DATA AND METHODS

The population-based survey data used in this analysis are from three DHS (Ethiopia, Kenya and Malawi) and two AIDS indicator surveys (Tanzania and Uganda) conducted during 2003 and 2006.^8^^–^12 The ANC surveillance data for these five countries were obtained from available ANC sentinel surveillance surveys, conducted during the same time period.^13^^–^17 Sample sizes for all surveys are provided in table 1.

**Table 1:** Data sources

	DHS/AIS		ANC		Number of DHS/AIS clusters	Number of ANC sites	Number of DHS/AIS clusters within 15-km catchment area of ANC sites	Number of women and men within 15-km catchment area of ANC sites interviewed in DHS/AIS (unweighted)
	Number tested and interview (unweighted)	Year	Number	Year				
Ethiopia	10 573 (men = 4631; women = 5942)	2005	28 247	2005	540	88	165	4596 (m = 2069, w = 2527)
Kenya	5996 (m = 2723; w = 3273)	2003	10 616	2003	400	40	153	3409 (m = 1664, w = 1745)
Malawi	5136 (m = 2272; w = 2864)	2004	8953	2005	522	19	139	2155 (m = 1037, w = 1118)
Tanzania	10 747 (m = 4774; w = 5973)	2003–4	17 813	2003–4	400	59	71	2849 (m = 1279, w = 1570)
Uganda	16 552 (m = 7476; w = 9376)	2004–5	9668	2005	417	19	111	5246 (m = 2429, w = 2817)

### Demographic and health surveys

The DHS/AIS surveys carried out in each of the five countries were designed to obtain national and regional estimates of HIV prevalence and associated sociodemographic and behavioural indicators among women and men. The DHS/AIS sample sizes take into account the estimated national HIV prevalence in each country, expected non-response rates for men and women, as well as design effects and expected confidence intervals.

The DHS/AIS surveys also routinely collect latitude and longitude coordinates for the communities where the survey respondents live.^18^ One location is recorded for each primary sampling unit in the sample. In order to maintain confidentiality of the survey respondents, these locations are offset randomly by a maximum of 2 km in urban areas, and 5 km in rural areas.

### Antenatal care surveillance surveys

ANC surveillance systems have been in place for a number of years in all five countries included in this analysis. In each country, the ANC surveillance estimates available for a time period closest to the DHS/AIS survey were used for this analysis. These data collection systems provide regular information to monitor HIV prevalence. ANC surveillance data from the five countries in this analysis followed the methodology described in the WHO guidelines.^19^

### Geographical information system methods

A geographical information system (GIS)-based methodology was used to identify the DHS/AIS clusters that were located within a reasonable distance of the ANC sites. Sample households within these clusters were expected to represent the catchment population of the ANC site.

A list of ANC surveillance facilities was obtained from the published sentinel surveillance reports for each country. Locations of the health facilities were georeferenced to the town or village where the site was located or the facility itself. In Ethiopia, the locations of the health facilities were provided by the Ministry of Health. The locations of ANC sites in Tanzania were georeferenced to corresponding towns and villages from the WHO/HealthMapper database. Missing facilities were matched to town or village locations manually, or by obtaining global positioning system (GPS) coordinates in collaboration with the national AIDS control programme. In Malawi, sentinel sites were matched to the facility GPS locations from the Ministry of Health Update of the Census of Health Facilities. In Uganda, the sentinel sites were located in the WHO/HealthMapper database (version 4.2),^20^ and were updated in collaboration with the Ministry of Health. The ANC sites in Kenya were georeferenced by matching the sentinel sites to the list of health facilities in the KEMRI/Wellcome Trust database and the WHO/Service Availability Mapping database.^21^ ^22^ All coordinates were projected to corresponding Universal Transverse Mercator (UTM) zones for each country.

**Figure 1:**
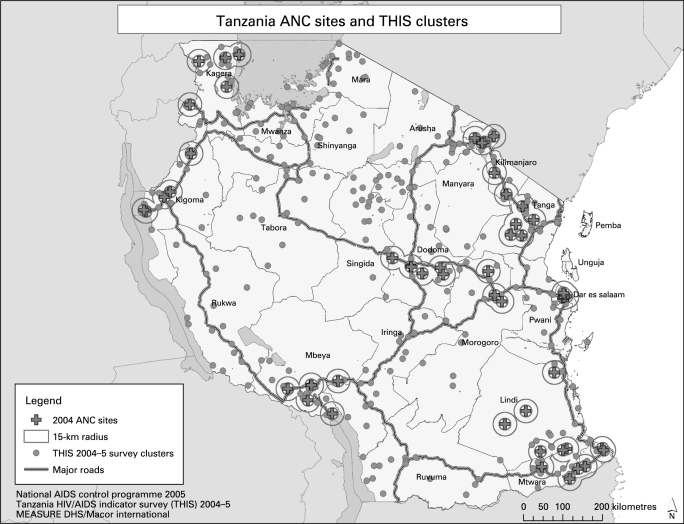
Tanzania HIV/AIDS survey clusters and antenatal care (ANC) surveillance sites.

The georeferenced locations of the ANC surveillance sites were then plotted with the DHS/AIS cluster locations. The distance from each DHS/AIS cluster to the nearest ANC site was calculated in kilometres as euclidian distance using ArcView 9.1.^23^ For each ANC site, the DHS clusters within 15 km were identified. The 15-km radius was used as an approximation of the geographic catchment area of the ANC site. The DHS/AIS sample clusters typically follow the distribution of the population in the country. The distribution of ANC sites in Tanzania illustrates the common scenario whereby the ANC sites are unevenly distributed across the country, and are typically located near major roads or towns (fig 1).

After identifying the DHS/AIS clusters within 15 km of an ANC site in each country, HIV prevalence estimates for women and men age 15–49 residing in the 15-km catchment areas of ANC sites were compared with ANC surveillance estimates for women age 15–49. DHS/AIS survey estimates for women were tabulated by current pregnancy status, experience of birth in past three years and whether attended ANC for the last birth. Comparisons were also made by broad age groups, urban/rural residence and educational status.

In Ethiopia and Tanzania, younger women (age 15–24) in the ANC catchment areas ofHIV prevalence estimates for the ANC surveillance data were taken from published surveillance reports cited previously. These estimates represent the unadjusted average prevalence for the total ANC surveillance sample. Because the ANC surveillance sites are purposively selected, and represent convenience samples of pregnant ANC attenders without a known probability of selection, it is not possible to calculate standard errors or meaningful confidence intervals for these estimates. Other research has suggested that plausibility bounds could be considered instead of confidence intervals for ANC estimates; these bounds range from plus or minus 3–4% depending on the stage of the epidemic and the strength of the surveillance system.^24^ ^25^ DHS/AIS estimates are presented with 95% confidence intervals for comparison purposes. An ANC estimate that falls within the 95% confidence interval of the DHS/AIS estimate is not considered to be significantly different from the DHS/AIS estimate.

## RESULTS

In four of the five countries in this analysis, national DHS/AIS surveys estimated HIV prevalence among women age 15–49 to be lower compared to HIV prevalence based on ANC surveillance data (table 2). In Ethiopia, the ANC estimate was about three times the estimate obtained in the DHS. The ANC estimates were also higher for Malawi and Tanzania than the DHS/AIS estimates, but in Uganda the ANC surveillance survey estimated HIV prevalence at 6.0%, lower than the DHS estimate at 7.5%. Comparing ANC and DHS/AIS estimates in urban and rural areas revealed similar patterns, in that HIV prevalence was higher in urban areas in all countries in both the data sources.

**Table 2:** Comparison of HIV prevalence among women age 15–49 in ANC sentinel surveillance and among women and men in DHS/AIS surveys who live in a community within 15 km from the nearest ANC surveillance site, 2003–5

	Women in ANC sentinel survey	Women and men in DHS/AIS survey* who live in a community within 15 km from the nearest ANC site			All women and men in DHS/AIS survey†; all women in ANC sentinel survey									
		Women	Men	Total	Urban			Rural			Total			
					DHS/AIS		ANC	DHS/AIS		ANC	DHS/AIS			
					Women	Men	Women	Women	Men	Women	Women	Men	Total	ANC
Ethiopia	5.3	4.3	2.5	3.6	7.7	2.4	9.5	0.7	0.7	2.2	1.9	0.9	1.4	5.3
		(3.4 to 5.3)	(1.7 to 3.4)	(3.0 to 4.2)	(6.4 to 9.0)	(1.5 to 3.3)		(0.4 to 0.9)	(0.4 to 1.0)		(1.5 to 2.2)	(0.7 to 1.2)	(1.2 to 1.7)	
Kenya	9.4	9.8	4.6	7.4	12.3	7.6	11.0	7.5	3.6	8.9	8.7	4.6	6.7	9.4
		(8.1 to 11.5)	(3.3 to 5.9)	(6.3 to 8.5)	(10.2 to 14.3)	(5.7 to 9.4)		(6.4 to 8.6)	(2.7 to 4.4)		(7.7 to 9.6)	(3.8 to 5.3)	(6.1 to 7.4)	
Malawi	16.9	18.5	12.8	15.9	18.0	16.3	20.4	12.5	8.8	13.0	13.3	10.2	11.8	16.9
		(15.7 to 21.3)	(10.1 to 15.5)	(14.0 to 17.9)	(14.1 to 21.9)	(12.4 to 20.2)		(11.2 to 13.8)	(7.5 to 10.1)		(12.1 to 14.6)	(9.0 to 11.5)	(10.9 to 12.7)	
Tanzania	8.7	8.6	7.6	8.2	12.0	9.6	11.4	5.8	4.8	3.4	7.7	6.3	7.0	8.7
		(7.0 to 10.2)	(5.8 to 9.4)	(7.0 to 9.4)	(10.4 to 13.6)	(7.8 to 11.3)		(5.1 to 6.5)	(4.1 to 5.5)		(7.0 to 8.4)	(5.6 to 7.0)	(6.5 to 7.5)	
Uganda	6.0	8.0	3.9	6.2	12.8	6.7	7.6	6.5	4.8	5.3	7.5	5.1	6.4	6.0
		(6.9 to 9.1)	(3.0 to 4.8)	(5.5 to 7.0)	(11.2 to 14.4)	(5.3 to 8.1)		(6.0 to 7.1)	(4.2 to 5.3)		(7.0 to 8.0)	(4.5 to 5.5)	(6.0 to 6.8)	

ANC, antenatal care; DHS, demographic and health surveys; AIS, AIDS indicator surveys.

(95% CI), *unweighted, †weighted.

In all five countries, HIV prevalence was higher among women who lived in a community within 15 km of the nearest ANC surveillance site than among all women included in the DHS/AIS survey, though this difference was only significant in Malawi (table 2). In three of the five countries, Kenya, Malawi and Uganda, the DHS/AIS estimate of HIV prevalence among women in the ANC catchment areas was greater (though not significantly) than the estimate from the ANC surveillance surveys. In Tanzania, the AIS estimate in the 15-km catchment area of the ANC sites was about the same (8.6%) as the ANC estimate (8.7%). In the fifth country, Ethiopia, the DHS estimate in the ANC catchment areas was much closer (4.3%) to the ANC surveillance survey estimate (5.3%) than the DHS national estimate for all women (1.9%), though these differences were not significant. However, in all five countries, HIV prevalence among men who lived in a community within 15 km of the nearest ANC surveillance site was lower (significantly in three countries) than among women in the ANC surveillance survey. In four countries, HIV prevalence among men and women in nearby clusters was lower than the ANC prevalence, though the difference was only significant in two countries.

In all five countries, women who were pregnant at the time of the DHS/AIS survey did not have significantly lower HIV prevalence than those who were not pregnant (table 3). HIV prevalence was significantly lower among women who gave birth in the three years preceding the DHS/AIS survey than among those who did not in three countries. However, there was no clear pattern in prevalence among women who received ANC for the last birth. In Kenya, HIV prevalence was significantly higher among women who gave birth in the last three years and received ANC for their last birth than among women who did not receive ANC or did not give birth in the last three years. But in the other three countries, Malawi, Tanzania and Uganda, the pattern was reversed.

**Table 3:** Comparison of HIV prevalence among women age 15–49 in ANC sentinel surveillance and in DHS/AIS surveys by pregnancy status, recent birth experience, and receiving antenatal care for last birth, 2003–5

	ANC	DHS/AIS			
	%	All women (weighted)		Women who live in a community within 15 km from the nearest ANC site (unweighted)	
		% (95% CI)	No	% (95% CI)	No
**Ethiopia**					
Total	5.3	1.86 (1.5 to 2.2)	5736	4.34 (3.4 to 5.3)	1911
Currently pregnant					
No		1.93 (1.6 to 2.3)	5256	4.49 (3.5 to 5.4)	1810
Yes		1.14 (0.2 to 2.1)	480	1.98 (0 to 4.7)	101
Gave birth in last 3 years					
No		1.96 (1.5 to 2.4)	3308	4.74 (3.6 to 5.9)	1417
Yes		1.73 (1.2 to 2.3)	2428	3.24 (1.7 to 4.9)	494
Attended ANC for last birth in last 3 years					
No ANC/no birth in last 3 years		1.63 (1.3 to 2.0)	5104	4.05 (3.1 to 5.0)	1681
Birth in last 3 years with ANC		3.74 (2.2 to 5.2)	631	6.52 (3.3 to 9.7)	230
**Kenya**					
Total	9.4	8.68 (7.7 to 9.6)	3151	9.76 (8.1 to 11.5)	1178
Currently pregnant					
No		8.81 (7.8 to 9.8)	2891	9.82 (8.1 to 11.5)	1100
Yes		7.3 (4.2 to 10.4)	260	8.97 (2.5 to 15.5)	78
Gave birth in last 3 years					
No		8.82 (7.6 to 10.1)	1961	9.96 (7.9 to 12.0)	823
Yes		8.46 (6.9 to 10.0)	1190	9.3 (6.3 to 12.3)	355
Attended ANC for last birth in last 3 years					
No ANC/no birth in last 3 years		8.56 (7.4 to 9.7)	2081	9.82 (7.8 to 11.8)	855
Birth in last 3 years with ANC		8.92 (7.2 to 10.6)	1070	9.6 (6.4 to 12.8)	323
**Malawi**					
Total	16.9	13.32 (12.1 to 14.6)	2686	18.48 (15.7 to 21.3)	736
Currently pregnant					
No		13.87 (12.5 to 15.2)	2323	19.03 (16.0 to 22.1)	636
Yes		9.78 (6.8 to 12.8)	362	15 (7.9 to 22.0)	100
Gave birth in last 3 years					
No		16.55 (14.6 to 18.5)	1,282	23.08 (18.9 to 27.3)	390
Yes		10.37 (8.8 to 11.9)	1404	13.29 (9.7 to 17.0)	346
Attended ANC for last birth in last 3 years					
No ANC/no birth in last 3 years		16.25 (14.3 to 18.2)	1337	22.81 (18.7 to 26.9)	399
Birth in last 3 years with ANC		10.41 (8.8 to 12.0)	1349	13.35 (9.7 to 17.0)	337
**Tanzania**					
Total	8.7	7.69 (7.0 to 8.4)	5753	8.63 (7.0 to 10.2)	1195
Currently pregnant					
No		7.8 (7.1 to 8.5)	5210	8.42 (6.9 to 10.0)	1117
Yes		6.77 (4.7 to 8.8)	533	11.69 (4.3 to 19.0)	77
Gave birth in last 3 years					
No		9.05 (8.1 to 10.0)	3206	9.52 (7.5 to 11.6)	777
Yes		5.98 (5.1 to 6.9)	2547	6.94 (4.5 to 9.4)	418
Attended ANC for last birth in last 3 years					
No ANC/no birth in last 3 years		8.72 (7.8 to 9.6)	3558	9.47 (7.5 to 11.5)	813
Birth in last 3 years with ANC		6.03 (5.1 to 7.0)	2195	6.81 (4.3 to 9.3)	382
**Uganda**					
Total	6.0	7.47 (7.0 to 8.0)	9350	8.02 (6.9 to 9.1)	2371
Currently pregnant					
No		7.66 (7.1 to 8.2)	8250	7.95 (6.8 to 9.1)	2125
Yes		6.47 (5.0 to 7.9)	1068	8.66 (5.0 to 12.3)	231
Gave birth in last 3 years					
No		8.48 (7.8 to 9.3)	4854	9.2 (7.7 to 10.8)	1392
Yes		6.37 (5.7 to 7.1)	4496	6.23 (4.7 to 7.7)	979
Attended ANC for last birth in last 3 years					
No ANC/no birth in last 3 years		8.46 (7.8 to 9.3)	5484	9.08 (7.7 to 10.6)	1509
Birth in last 3 years with ANC		6.05 (5.3 to 6.8)	3866	6.03 (4.4 to 7.6)	862

DHS/AIS samples had significantly lower HIV prevalence than younger women in the ANC surveillance surveys (table 4). This pattern reversed for older age groups, where women age 25 and older in the ANC catchment areas of DHS/AIS surveys had higher HIV prevalence than those in the ANC surveillance surveys. This finding suggests that women covered by ANC surveillance sites are not representative of all women even within the 15-km catchment areas of the surveillance sites. However, this differential age pattern in HIV prevalence between the two data sources is insignificant when a comparison is made with women from the DHS/AIS surveys who lived in the 15-km catchment areas of the ANC surveillance sites and received ANC for their last birth in the three years preceding the survey.

**Table 4:** Comparison of HIV prevalence among women age 15–49 from ANC sentinel surveillance and the DHS/AIS survey respondents residing within 15 km of an ANC site who gave birth in last three years and attended ANC in a public facility by age, residence, and education, 2003–5 (unweighted)

	Ethiopia			Kenya			Malawi			Tanzania			Uganda		
	DHS/AIS 15-km catchment area	Attended ANC DHS/AIS 15-km catchment area	ANC	DHS/AIS 15-km catchment area	Attended ANC DHS/AIS 15-km catchment area	ANC	DHS/AIS 15-km catchment area	Attended ANC DHS/AIS 15-km catchment area	ANC	DHS/AIS 15-km catchment area	Attended ANC DHS/AIS 15-km catchment area	ANC	DHS/AIS 15-km catchment area	Attended ANC DHS/AIS 15-km catchment area	ANC
Total	4.3	6.5	5.3	9.8	9.6	9.4	18.5	13.4	16.9	8.6	6.8	8.7	8.0	6.0	6.0
	(3.4 to 5.3)	(3.3 to 9.7)		(8.1 to 11.5)	(6.4 to 12.8)		(15.7 to 21.3)	(9.7 to 17.0)		(7.0 to 10.2)	(4.3 to 9.3)		(6.9 to 9.1)	(4.4 to 7.6)	
**Age**															
15–24	2.5	7.7	5.6	5.8	6.4	NA	11.8	10.2	14.3	2.6	2.8	7.4	4.5	6.5	5.0
	(1.5 to 3.6)	(1.6 to 13.7)		(3.8 to 7.8)	(2.3 to 10.5)		(8.3 to 15.4)	(5.3 to 15.2)		(1.2 to 4.0)	(0.1 to 5.5)		(3.2 to 5.7)	(3.9 to 9.2)	
25–34	6.0	6.0	5.4	14.7	13.2	NA	24.7	16.8	21.2	12.9	9.4	11.0	11.4	6.6	7.9
	(4.0 to 8.1)	(1.6 to 10.3)		(11.2 to 18.3)	(7.6 to 18.8)		(19.2 to 30.2)	(10.5 to 23.1)		(9.6 to 16.1)	(5.1 to 13.8)		(9.2 to 13.6)	(4.2 to 9.0)	
35–49	6.0	5.7	3.3	10.4	7.7	NA	22.2	13.2	16.9	13.2	8.8	6.7	10.1	2.6	4.0
	(3.8 to 8.1)	(0 to 13.8)		(6.8 to 13.9)	(0 to 16.4)		(16.0 to 28.4)	(3.8 to 22.6)		(9.2 to 17.2)	(1.2 to 16.3)		(7.5 to 12.7)	(0 to 5.6)	
**Residence**															
Urban	5.9	7.4	9.5	12.5	14.2	11.0	20.6	19.5	18.3	11.0	9.6	11.4	12.3	11.3	7.6
	(4.6 to 7.2)	(3.2 to 11.7)		(9.9 to 14.9)	(8.8 to 19.6)		(15.2 to 26.1)	(11.0 to 28.0)		(8.7 to 13.3)	(5.5 to 13.7)		(10.5 to 14.2)	(8.1 to 14.5)	
Rural	1.5	4.9	2.2	6.4	5.0	8.9	17.6	11.2	13.0*	5.1	3.8	3.4	3.3	2.0	5.3
	(0.6 to 2.4)	(0.1 to 9.6)		(4.3 to 8.5)	(1.6 to 8.4)		(14.3 to 20.9)	(7.3 to 15.1)		(3.2 to 7.1)	(1.0 to 6.6)		(2.2 to 4.3)	(0.8 to 3.3)	
**Education**															
None	3.2	3.1	NA	5.8	NA	6.0	21.3	12.7	17.9	8.2	3.5	5.2	7.2	6.8	3.9
Primary	(1.9 to 4.4) 4.6	(0 to 6.5) 6.7	NA	(0.8 to 10.9) 11.7	11.4	10.6	(14.8 to 27.8) 17.2	(4.7 to 20.6) 12.3	16.1	(4.0 to 12.3) 9.0	(0 to 8.3) 7.2	9.3†	(4.5 to 9.9) 8.4	(2.7 to 10.8) 5.5	4.8
	(2.6 to 6.5)	(0 to 14.2)		(9.1 to 14.3)	(6.9 to 15.9)		(13.7 to 20.7)	(7.9 to 16.7)		(7.0 to 10.9)	(4.2 to 10.2)		(6.8 to 9.9)	(3.5 to 7.5)	
Secondary+	5.5	10.3	NA	8.0	7.9	9.2‡	20.2	19.6	33.3	7.6	9.1	NA	7.8	6.7	6.1
	(3.8 to 7.1)	(3.8 to 16.9)		(5.6 to 10.5	(2.9 to 12.9)		(13.1 to 27.2)	(7.7 to 31.5)		(3.9 to 11.4)	(0 to 19.4)		(6.0 to 9.7)	(3.4 to 10)	

*Includes urban and semi-urban.

†Some education is the only category in the report.

‡Prevalence for secondary+ computed by taking a weighted average of “secondary” and “higher” categories.

The total ANC prevalence estimates are generally closer to the urban rather than the rural ANC estimates in Tanzania and Malawi, suggesting some over-representation of urban women in these ANC surveillance surveys. In both the ANC surveillance surveys and the ANC catchment areas of the DHS/AIS surveys, urban women have higher HIV prevalence than rural women, but there are no consistent patterns in the urban/rural differential between the two data sources. By education categories also there are no significant patterns within or between the two data sources.

## DISCUSSION

The study found that in four of the five countries, Ethiopia, Kenya, Malawi, and Tanzania, the national DHS/AIS estimates were lower than the ANC surveillance estimates. In Uganda, where the epidemic is believed to have stabilised or levelled,^26^ ^27^ the ANC surveillance estimate was slightly lower than the DHS/AIS estimates.

In all five countries, HIV prevalence was higher among women who lived in a community within 15-km of the nearest ANC surveillance site than among all women included in the DHS/AIS survey. This may be because ANC sites tend to be disproportionately located near urban areas where HIV prevalence is higher.

When the ANC surveillance estimates were compared with the DHS/AIS estimates for women residing in the 15-km catchment areas of the ANC surveillance sites, the DHS/AIS estimates were about the same or higher in four of the five countries; and in the fifth country, Ethiopia, the gap between the two estimates was considerably narrowed. This suggests that the two data sources compare rather well when the comparison is restricted to women living in the catchment areas of the ANC surveillance sites. However, in all countries, HIV prevalence among men living in the catchment areas of ANC surveillance sites was much lower than HIV prevalence among women in ANC surveillance surveys.

Prevalence among men residing within 15 km of the catchment area of an ANC site was lower than HIV prevalence among women in the ANC sample in every country. Prevalence among men and women combined in the 15-km catchment area of ANC sites was lower than the prevalence among ANC attenders in all countries but Uganda. In Malawi and Uganda, the prevalence among men and women in the nearby clusters was closer to the ANC prevalence than the prevalence among women only.

In all countries with available data, ANC estimates were higher for younger women (15–24) than the DHS/AIS estimates for younger women in the catchment areas, but lower at older ages. This finding suggests that women covered by ANC surveillance sites are not representative of all women even within the 15-km catchment areas of the surveillance sites. The total ANC prevalence estimates were generally closer to the urban ANC estimates, again reflecting a possible urban bias in the ANC surveillance estimates. The urban prevalence was higher than rural prevalence in all countries both in the ANC and the DHS/AIS surveys, but there were no consistent patterns in education differentials between the two data sources.

Some limitations of this analysis should be kept in mind when interpreting the findings. A major limitation is that the selection of DHS/AIS clusters within a 15-km radius around the ANC surveillance sites is based on the assumption that 15 km is a reasonable distance which most women would travel for ANC care, which may not reflect a true catchment area for an ANC site. However, a previous analysis of ANC attendees at sentinel surveillance sites in Uganda showed that these distances corresponded reasonably well with the actual administrative areas where clients were living.^16^ For a more meaningful comparison, the catchment areas should be defined by examining the ANC client records for each surveillance site.

Another source of bias may be due to displacement of GPS coordinates of DHS/AIS clusters to protect confidentiality of survey participants. However, because the displacement was random and the results from individual ANC catchment areas were aggregated up to the national level, any effect of such bias is expected to be small.

The DHS/AIS samples may also be biased owing to differential non-response in the surveys, as well as exclusion of non-household population groups. However, an analysis of effects of non-response and exclusion of non-household population on national HIV prevalence estimates in the DHS/AIS surveys in several countries has shown that the impacts of such bias tend to be small and insignificant.^28^

Despite these limitations, the findings of this study suggest that HIV prevalence estimates derived from ANC sentinel surveillance surveys tend to overestimate HIV prevalence among women in the general population. However, the DHS/AIS estimates of HIV prevalence among women compare well with the ANC surveillance estimates when the comparison is restricted to women residing within the catchment areas of the ANC surveillance sites. Patterns by age and urban/rural residence point to possible sources of bias in the ANC estimates. The study reinforces the need to evaluate HIV prevalence estimates for potential sources of bias, and suggests that HIV prevalence data from population-based surveys can be used to calibrate estimates from clinic-based surveillance.

**Key messages**

HIV prevalence estimates from population-based surveys tend to be lower than HIV prevalence estimates from antenatal care (ANC) sentinel surveillance data. The study demonstrates that restricting the population sample to the geographical areas near HIV sentinel surveillance sites yields similar HIV prevalence estimates, and highlights important differences in the two populations. The findings help to quantify and understand the differences between population-based and ANC sentinel surveillance HIV prevalence estimates.
